# Multi-responsive hydrogels for drug delivery and tissue engineering applications

**DOI:** 10.1093/rb/rbu006

**Published:** 2014-10-20

**Authors:** Jennifer M. Knipe, Nicholas A. Peppas

**Affiliations:** ^1^Department of Chemical Engineering, C0400, The University of Texas at Austin, Austin, TX 78712, USA, ^2^Department of Biomedical Engineering, C0800, The University of Texas at Austin, Austin, TX 78712, USA, ^3^College of Pharmacy, C0400, The University of Texas at Austin, Austin, TX 78712, USA

**Keywords:** hydrogels, intelligent polymers, regenerative medicine, drug delivery

## Abstract

Multi-responsive hydrogels, or ‘intelligent’ hydrogels that respond to more than one environmental stimulus, have demonstrated great utility as a regenerative biomaterial in recent years. They are structured biocompatible materials that provide specific and distinct responses to varied physiological or externally applied stimuli. As evidenced by a burgeoning number of investigators, multi-responsive hydrogels are endowed with tunable, controllable and even biomimetic behavior well-suited for drug delivery and tissue engineering or regenerative growth applications. This article encompasses recent developments and challenges regarding supramolecular, layer-by-layer assembled and covalently cross-linked multi-responsive hydrogel networks and their application to drug delivery and tissue engineering.

## Introduction

Biomaterials are materials used in the presence of physiological conditions, either as an implant or any other device that will interact with biological fluids [1]. The field of biomaterials involves not only the design of a structurally and immunologically sound material, but also attempts to create a biomimetic system that can interact with the biological environment through various cellular and physiological cues [2, 3]. Cellular interactions on the molecular scale are fundamental to the design of any material to be used in the body. Hydrogels are well-suited as biomaterials due to their molecular structure and inherent ability to imbibe physiological fluids [4].

In recent years, we have seen steady expansion of research utilizing ‘intelligent’ or stimuli-responsive hydrogels, particularly in the fields of drug delivery and tissue engineering [5]. Stimuli-responsive hydrogels are cross-linked, hydrophilic polymer networks that undergo a physicochemical transition in response to a change in external stimuli such as pH, temperature, light and analyte concentration, to name a few [6–8]. The response to stimuli often manifests as a change in surface charge or hydrophobicity, change in phase volume of the gel, breaking of bonds resulting in degradation or gel–sol transition, or a combination of these [6, 9]. Gil and Hudson reviewed an extensive list of stimuli-responsive polymers, as well as bioconjugates made with the polymers, including the design criteria taken into consideration to achieve desired responses useful for biomedical applications [10].

Hydrogels with specific, tunable and even reversible responses to environmental stimuli have been known for decades as excellent candidates for drug delivery [7, 11] and regenerative medicine [12, 13] applications. Peppas *et al.* have dedicated their careers to developing complexation and responsive hydrogels suitable for delivery of delicate therapeutics [14–18]. Likewise, innumerable researchers have used chitosan [19, 20], poly(*N-*isopropyl acrylamide) [21] and various other natural and synthetic responsive polymers [22] in the areas of tissue engineering and regenerative medicine for many decades.

However, the need for materials with both broad utility and increased specificity to the application is ever-present. The fourth-generation biomaterials known as ‘smart’ or biomimetic materials that respond to the host environment are evolving into sophisticated materials that respond to multiple stimuli in order to better mimic biological processes [23]. Multi-responsive hydrogels are a prime example of these advanced systems. These composite, interpenetrating network or supramolecular hydrogels respond to two or more environmental stimuli, including pH, temperature, photons, enzymes, redox potential, electric or magnetic field, or analyte concentration. The output of these functional materials is contraction, swelling, degradation, color change, etc., and the exact response may be manipulated by tuning the polymer chemistry. This has tremendous advantages in terms of physiological compatibility, targeted drug delivery [24], controlled release, and directed cell growth [25].

Herein, we review recent developments in the composition, functional attributes and biomedical applications of multi-responsive hydrogels.

## Supramolecular and *In Situ-*Forming Hydrogels

‘Click’ chemistries and controlled polymerization methods such as reversible addition fragmentation chain transfer (RAFT) and atom transfer radical polymerization (ATRP) have enabled the challenging synthesis of block copolymers, dendrimers, synthetic polypeptides and other macromolecules that have domains or side groups with multiple unique and even opposing properties [26, 27]. The responsive domains of these copolymers can undergo intermolecular interactions upon exposure to environmental stimuli, resulting in spontaneous self-assembly into supramolecular assemblies such as micellar or fibrillar structures [28]. Supramolecular hydrogels are cross-linked by hydrogen bonding, ionic bonds, van der Waals forces and hydrophobic interactions [29]. Host–guest chemistry, in which two or more molecules specifically and selectively bind in a non-covalent manner [30, 31], is also prevalent in supramolecular hydrogels. Although the use of supramolecular hydrogels in biomedical applications has recently been reviewed elsewhere [29], a few examples of their multi-responsive functionality will be discussed here.

β-Cyclodextrin (β-CD), a biopolymer widely used in the pharmaceutical and food industries, serves as a host molecule during self-assembly by forming a cavity in which the guest molecule binds, thereby forming an inclusion complex that cross-links the gel network [30, 32, 33]. As they are commercially available, nontoxic and contain nonpolar regions for binding, β-CD is one of the most commonly used host molecules in the synthesis of supramolecular hydrogels, though molecules such as crown ethers, calixarenes and cucurbiturils are also used to some extent [28, 32]. β-CD is not stimuli-responsive, but it can be used in conjunction with responsive guest molecules to achieve sol–gel transition due to change in environmental conditions. Indeed, researchers have incorporated cyclodextrins with pH- or T-responsive structure to achieve dual action [34]. Guan *et al.* [35] developed a supramolecular hydrogel via host–guest interaction between poly(N-isopropyl acrylamide) (PNIPAAm) chains modified with azobenzene groups and CD dimers containing disulfide bonds. PNIPAAm is known to be a temperature-sensitive polymer, and the host–guest interaction was also light-sensitive due to the azobenzene groups. Additionally, the disulfide groups were susceptible to cleavage by reduction; since reductive environments are common in living organisms, this third response could be advantageous to certain biomedical applications such as intracellular delivery of therapeutics.

To create materials with better biomimetic responses and biocompatibility in combination with reproducibility and stability, many researchers are incorporating peptides into polymeric systems [27, 36, 37]. These hybrid macromolecules are capable of self-assembly into organized structures such as micelles or thin films, which are useful in drug delivery and tissue engineering applications [38]. Peptide incorporation within polymer systems also provides the opportunity for enzyme-triggered responses, which are highly selective and efficient [39]. In one such case, researchers positioned a peptide between a hydrophilic and thermosensitive block of a triblock copolymer [36]. The polymers self-assemble into micellar structures above the cloud point of the P(NIPAAm) in aqueous solutions. Collagenase, a model enzyme for inflamed tissue metalloproteases, was able to cleave the peptide blocks, resulting in a ‘shedding’ of the hydrophilic corona of the supramolecular assembly. The system has potential for thermoresponsive and enzymatically degradable drug delivery.

**Figure 1.**
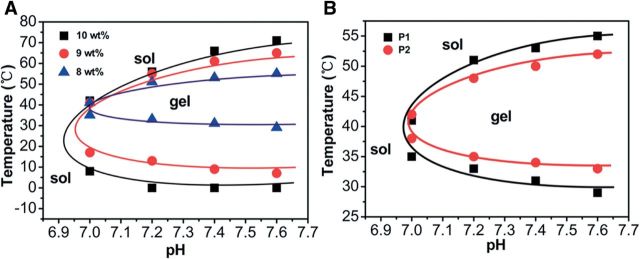
Sol–gel phase diagrams of poly(ether-urethane) copolymers: (A) effect of concentration on sol–gel phase diagram of poly(ether-urethane) copolymer with 1:6 poly(ethylene glycol):(2,2′-dithiodiethanol + N-methyldiethanolamine) (P1); (B) effect of composition on sol–gel phase diagram poly(ether-urethane) copolymer with 1:6 (P1) and 1:4 (P2) poly(ethylene glycol):(2,2′-dithiodiethanol + N-methyldiethanolamine) at a concentration of 8 wt%. Reprinted with permission from Li X, Wang Y, Chen J *et al.* Controlled release of protein from biodegradable multi-sensitive injectable poly(ether-urethane) hydrogel. *ACS Appl Mater Interf* 2014;**6**:3640–7. doi: 10.1021/am405927f. Copyright 2014 American Chemical Society [44].

Supramolecular and other *in situ-*forming hydrogels are particularly interesting due to their rheological versatility; in sol form, they may possess a viscosity suitable for injection-based treatments, followed by gel-phase transition upon exposure to physiological conditions [40]. This feature has been employed by a number of research groups to create injectable systems for site-specific drug delivery [41–43] and regenerative medicine [40] that can overcome clogging of the needle during injection. Recently, Li *et al.* [44] synthesized a series of poly(ether-urethane)-based polymers with varying poly(ethylene glycol) and *N-*methyl diethanolamine (MDEA) ratios. The temperature response of the polymer was modulated by varying the incorporation of the pH-sensitive MDEA such that it remained in a sol state under injection conditions but gelled in physiological conditions, as shown in Fig. 1. Disulfide bonds were incorporated via 2,2-dithiodiethanol to allow degradation by a reductive environment. The researchers demonstrated *in vivo* gel formation following subcutaneous injection in rats as well as *in vitro* release of insulin in response to degradation by glutathione over 28 days.

In another interesting study, a polyacrylamide backbone was grafted with photoresponsive thymine derivatives, and upon exposure to 270 nm UV light, the thymine underwent dimerization causing a sol–gel transition in the polymer that could be reversed with exposure to 240 nm UV light [45]. As a secondary response, the polymer can also bind with Hg^2+^ to form a gel structure that is reversible in the presence of thiol-containing molecules. The gel was able to release dye-doped nanoparticles following either a change in pH or/and increased concentration of thiol-containing molecules, making it a good candidate for both controlled-release and sensitive detection of thiol-containing molecules. However, the binding with Hg^2+^ is an obvious concern for biomedical applications as this may have some inherent toxicity.

Mikos and collaborators have been developing PNIPAAm-based hydrogels capable of both thermogelation and amine:epoxy chemical cross-linking to achieve injectable *in situ-*forming hydrogels for cell delivery [46]. These gels rapidly form physical cross-links following injection at physiological temperature due to PNIPAAm thermogelation, whereas the chemical cross-linking between glycidyl methacrylate and diamine-functionalized polyamidoamine proceeds more slowly. The lower critical solution temperature (LCST) of the polymer was tuned by incorporation of acrylic acid to be between room and physiological temperature. Additionally, a hydrolyzable lactone ring was incorporated to allow degradation of the gel over a period of about 30 days. Owing to its *in situ* thermogelation, biodegradability and ability to encapsulate mesenchymal stem cells, this material shows excellent promise for cell delivery and regenerative medicine applications.

**Scheme 1.**
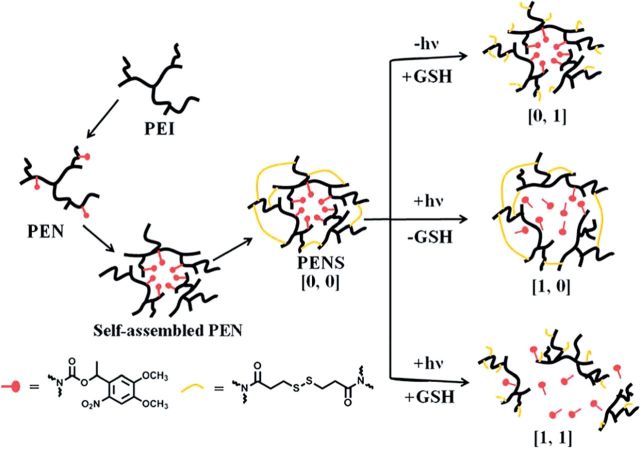
Schematic presentation of the assembly of the dual-responsive nitrobenzyl-modified-polyetherimide micelles (PEN) cross-linked with dithiodipropionic acid (PENS) and their response to light and/or a reductive environment [47].

In an effort to exercise greater control over the release of encapsulated cargo, Huang *et al.* developed a supramolecularly assembled nanoparticle system that exhibited the ‘AND’ logic response, which means response was triggered only when both stimuli were present simultaneously [47]. Polyetherimide was modified with a photo-cleavable nitrobenzyl derivative, resulting in amphiphilic polymers capable of micellar assembly in water. The micelles were then stabilized with a cross-linker-containing disulfide bonds. In this way, the micelles could only be disrupted if both the nitrobenzyl derivative was cleaved and the disulfide bonds reduce via simultaneous or sequential application of light and a reducing agent, shown in Scheme 1. As expected, the greatest release of the therapeutic cargo, doxorubicin, was demonstrated when both stimuli were present, though some release was observed in the presence of light alone.

## Layer-By-Layer Assembly

Layer-by-layer (LbL) assembly is simply the alternate adsorption of oppositely charged molecules onto a solid core [48], resulting in precise, multilayer, polyelectrolyte films or particles that can be incorporated with therapeutics for drug delivery or biosensory applications [49, 50]. Researchers have created LbL systems that are responsive to a number of stimuli, including light [51], pH [52, 53], and temperature [54–56], to name a few. Since LbL-assembled films inherently possess polyelectrolytes capable of eliciting a pH response, it is most interesting to explore the relationship between the polyelectrolyte and secondary responses and how they may be tuned for biomedical applications.

LbL assembly of microgel [56] or micellar [57] structures has also been demonstrated in the literature as a method to control responsive properties of the material. By varying the composition of the microgels, Clark and Lyon showed that the temperature response of the LbL-assembled film of P(NIPAAm-*co-N*-isopropyl methacrylamide) microgels with a polycationic layer could be tuned not only by the composition of the microgels, but also by the ratio of different microgel compositions assembled in the film [56]. The authors conclude that the microgels apparently behave independently of the polyelectrolyte matrix but acknowledge that more investigation is needed into the effect of the polycation layers on the temperature response of the microgels, suggesting that pH may also play a role in the temperature response or vice versa, as others have shown [44, 58–60].

Similarly, Yusan *et al.* [57] found that the upper critical solution temperature (UCST) response of the zwitterionic poly[3-dimethyl (methacryloyloxyethyl) ammonium propane sulfonate] (β-PDMA)-coronae of micelles assembled via LbL deposition into a multilayer film altered the effect of decreasing pH on the film thickness. The increase in thickness of the film at a temperature below the UCST was attributed to the phase separation of the β-PDMA chains, resulting in voids within the multilayers that increased the imbibition of water and volume of the film. Although a temperature response of the film was not explicitly shown, this effect of temperature on the pH response permits another degree of control over the stimuli response of the material, which is useful for functional surface or drug delivery applications.

## Cross-Linked Polymers and Interpenetrating Polymer Networks

‘Click’ chemistries have also been used to cross-link polymeric chains into a hydrogel network for biomedical applications. For example, thermoresponsive PNIPAAm was modified with various functionalities to serve as a cross-linker, then clicked with azido-grafted hyaluronic acid (HA) to create a polymer network [61]. The resulting hydrogel exhibited a distinct volume phase transition temperature (VPTT) between 32 and 34°C as well as pH-dependent swelling behavior. Importantly, the pore size of the gels could be controlled by varying the spacing of grafted azide functions on the HA chains or by varying the chain length of the PNIPAAm cross-links, which has great utility in designing scaffolds to mimic the extracellular matrix. Additionally, release of fluorescein isothiocyanate-labeled dextran from the gels was increased at temperatures above the VPTT, indicating potential dual use as a drug-releasing scaffold material. Degradability of the gels by hyaluronidase was found to be controlled by degree of cross-linking.

Although covalently cross-linked hydrogels have increased stability and mechanical integrity, their use in biomedical applications that require injections is limited, hence the drive to synthesize covalently cross-linked polymers that undergo a sol–gel transition in response to stimuli such as pH or temperature [62–64]. To this end, homopolymers and copolymers of 2-(2-methoxyethoxy) ethyl methacrylate (MEO_2_MA) and N,N-(dimethylamino) ethyl methacrylate (DMAEMA) were modified with tryptophan side–chains [62]. Owing to an indole moiety, the tryptophan side-chains introduced inherent fluorescent properties to the network. These polymer chains were able to form reversible, covalent imide bonds with 4-formylphenyl 40-formylbenzoate in the presence of glacial acetic acid, resulting in a cross-linked network. Gelation of the polymer was dependent upon polymer and cross-linker concentrations, amino acid content within the polymer, and solution pH. The gels also possessed an LCST that was independent of pH, but was affected by high amino acid content. The inherent fluorescence and ability to reshape the gel endow it with properties desirable for biomedical applications, particularly injectable cell and drug delivery.

**Figure 2.**
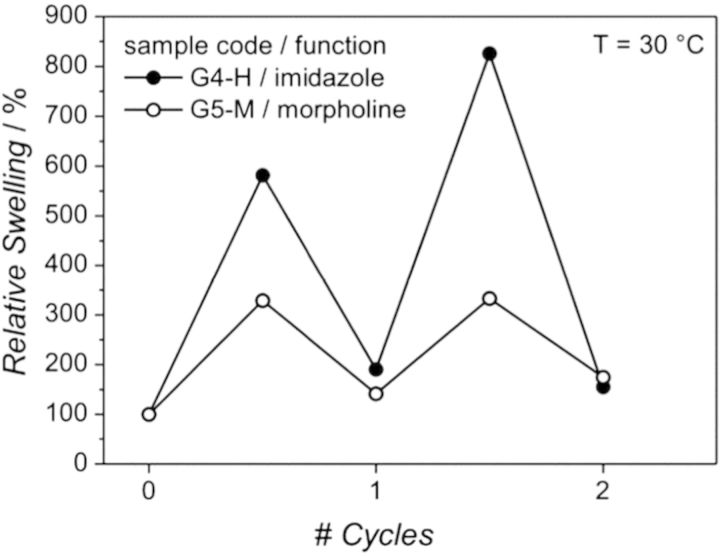
Reproducibility of relative swelling degrees of 20 wt% polymer precursor with imidazole (G4-H) and 15 wt% polymer precursor with morpholine (G5-M) gels after alternating exposure to carbon dioxide and nitrogen over two 1-hour cycles switching gases every 30 min at 30°C.Reproduced from Reinicke *et al.* [65] with permission from The Royal Society of Chemistry.

In a different approach, Reinicke *et al.* incorporated a thiolactone onto a linear polymer backbone [65]. The thiol ring may be opened in response to primary amines, serving as an *in situ* source for functionalization, modification or cross-linking via conjugation with the free thiol. Unexpectedly, in the presence of dichloromethane (DCM), the ring opening reaction resulted in covalent, non-degradable cross-linking of the polymer chains. Reaction time with DCM, polymer precursor concentration and thiolactone content were each varied to tune the degree of swelling of the gel. Upon functionalization with morpholine and imidazole moieties, the gels showed a response to carbon dioxide in the form of reversible swelling. Although the extent of swelling was not repeatable over multiple cycles, it did exhibit temperature dependence, approaching initial swelling ratios at temperatures >30°C as shown in Fig. 2. A solvochromic dye was incorporated into the gel via acrylate–thiol reaction to induce a reversible color change in response to temperature. However, the solvochromtic dye is most responsive at acidic conditions that are not physiologically relevant.

According to the International Union of Pure and Applied Chemistry (IUPAC) definition, interpenetrating polymer networks (IPNs) consist of two or more polymer networks that are not chemically cross-linked with each other, but are physically entangled such that they cannot be separated without breaking chemical bonds [66]. Similarly, a semi-IPN comprises one or more cross-linked polymer networks physically entangled with one or more linear or branched polymers [66]. An IPN exhibits the individual stimuli responses of its constituents with the additional possibility of increased mechanical integrity, tunable network properties and cell compatibility [67, 68]. Multi-responsive properties incorporated into IPNs include temperature response with biodegradability [60, 69, 70], pH-response and hydrophobicity [70], or the combination of temperature and pH response [71–75].

An IPN can be used to combine interesting properties of synthetic polymers, such as the swelling response of polyacrylamide (PAAm), with the biodegradability and biocompatibility of a biopolymer such as poly(γ-glutamic acid) (γ-PGA) [60]. The molar proportions of PAAm and γ-PGA were varied, resulting in temperature-dependent swelling that could be increased by incorporating more γ-PGA. Researchers attributed the temperature dependence to an increase in flexibility of the γ-PGA chains at higher temperatures due to breaking of hydrogen bonds, causing an increase in swelling ratios. Additionally, the pH dependence of γ-PGA caused higher swelling ratios as pH increased, particularly at pH values greater than the pKa of γ-PGA, ∼ 4.0–4.8. Also important to note was the rapidity of the swelling; all of the gel formulations reached the equilibrium swelling point in <40 min. This could be useful for drug delivery applications which require a fast release of the therapeutic.

## Application to Regenerative Medicine

### Drug delivery

**Figure 3.**
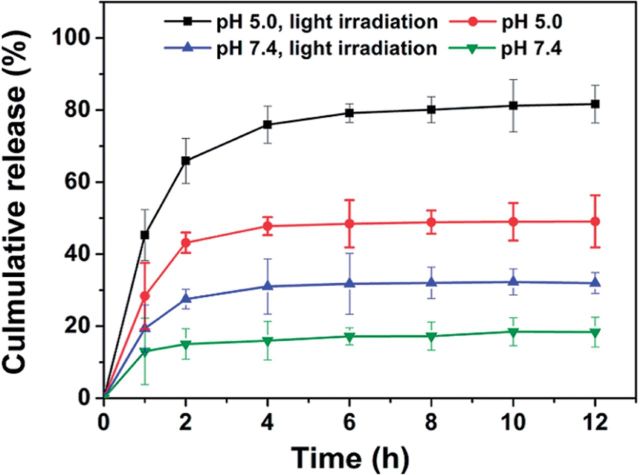
*In vitro* release of camptothecin from cross-linked polymeric micelles with a molar feed ratio of 0.23 of o-nitrobenzyl succinate to glucosamine units and a degree of substitution of 19.3.Release was evaluated at pH 5.0 or 7.4 with and without irradiation by 365 nm UV light for 10 min. Reprinted with permission from Meng L, Huang W, Wang D *et al.* Chitosan-based nanocarriers with ph and light dual response for anticancer drug delivery. *Biomacromolecules* 2013;**14**:2601–10. doi: 10.1021/bm400451v. Copyright 2013 American Chemical Society [76].

As externally triggered and spatiotemporal drug release mechanisms such as heat, magnetic fields and light are becoming more widely investigated for cancer treatments, so are polymer systems that take advantage of these mechanisms. One such system, comprising amphiphilic glycol chitosan modified with *o-*nitrobenzyl succinate (NBS) conjugates, incorporated responses to both internal and external stimuli [76]. The light-sensitive chains self-assembled into micelles in water and were stabilized by acid-labile cross-linking with glutaraldehyde. In acidic conditions, the imine bonds in the cross-links were cleaved and the chitosan was protonated, resulting in swelling of the nanocarriers and release of the drug. Following 10 min of external UV irradiation, the NBS groups were cleaved and no micelles were observed. It was demonstrated that the release of the cancer drug camptothecin was increased following exposure to UV light at both acidic pH and neutral pH relative to the non-UV controls. However, release of the drug was the highest at the low pH and UV exposure conditions, confirming that both the internal and external stimuli were necessary for optimal release, shown in Fig. 3.

**Figure 4.**
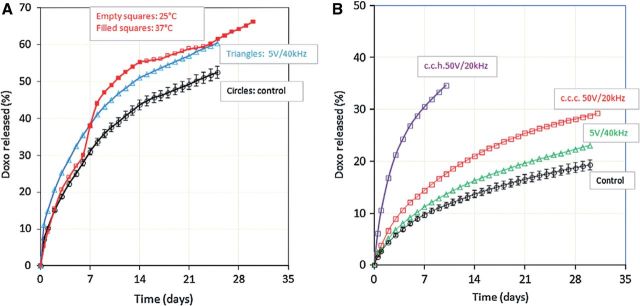
(A) Cumulative doxorubicin release (%) from loaded L-phenylalanine and N-isopropylacrylamide hydrogels with 2.0 ml magnetic particles in PBS pH 7.40: under AMF stimulation (5 V and 40 kHz at 25°C, blue triangles); control (25°C, black circles); change in temperature (25°C empty red squares, 37°C red filled squares); (B) cumulative doxorubicin release (%) from L-valine homopolymer hydrogel with magnetic particles under various AMF stimulation (PBS pH 7.40 at 25°C): control (black circles); stimulated by 5 V and 40 kHz (green triangles); stimulated by 50 V and 20 kHz with cell copper condenser (c.c.c., red squares); stimulated by 50 V and 20 kHz with copper coil honeycomb (c.c.h., violet squares) [77].

Multi-stimuli responsive hydrogels are instrumental in achieving site-specific delivery of chemotherapeutics to improve drug efficacy and limit toxic side-effects. Hydrogels based on the hydrophobic amino acid residues L-phenylalanine and L-valine were incorporated with magnetic nanoparticles for the site-specific delivery of the cancer therapeutic doxorubicin (DOX) [77]. These gels exhibited prolonged release of DOX in response to temperature, whereas the pH response affected loading and release efficiency of DOX due to electrostatic interactions between the drug and carboxyl groups on the amino acid residues. Additionally, the hydrogels could be externally stimulated by an alternating magnetic field to enhance the release of DOX, shown in Fig. 4. Owing to the release timescale of weeks and ability to remotely control DOX release, this system has potential to reduce total number of DOX administrations while achieving site-specific delivery to the cancer target.

Hydrogels incorporating multiple responses can also be useful in encapsulating and delivering multiple drugs with different properties. Lv *et al.* proposed selective release of one of multiple encapsulated molecules in response to a specific stimulus, i.e. releasing one payload in response to pH and one in response to chemical reduction [78]. This was achieved by using a redox and pH-sensitive polymer, polyaniline, to create nanocontainers that encapsulated the first molecule of interest. The nanocontainers were then decorated with gold nanoparticles that could be loaded with a second molecule. The molecule contained in the polymer shell was shown to be released only as a function of increasing pH, whereas the second molecule loaded onto the gold nanoparticles was released only as a function of concentration of reducing agent. Although the authors proposed this selective release system for anti-corrosion applications, it could easily be translated to biomedical applications such as drug delivery given the dynamic physiological pH environments and reductive intracellular environments.

### Closed-loop delivery systems

A multi-responsive system may be designed to not only sense an analyte, but also deliver a therapeutic in response to the analyte presence. As shown by Gu *et al.*, such a system can have important implications as a closed-loop treatment for diabetes [79]. Uniform chitosan microgels containing insulin and glucose oxidase and catalase nanocapsules were formed by a facile electrospray procedure. In the presence of hyperglycemic glucose concentrations, the microgels swelled significantly as the enzymatic nanocapsules converted glucose to gluconic acid, enabling the protonation and swelling of chitosan chains in the increasingly acidic solution. In these conditions, insulin was released steadily over about 3 h and was even shown to decrease blood glucose level in diabetic mice. The built-in delivery trigger via enzymatic nanocapsules certainly enhances the specificity of the response and has great potential for closed-loop delivery, but likely would not prevent the microgels from swelling in acidic conditions without the presence of glucose.

The transience of multi-responsive supramolecular hydrogels has also been used as a method of sensing small molecules, glucose [58, 80] being a molecule of great interest. Zhou *et al.* developed a system that underwent a sol–gel transition in response to temperature, oxidizing agent or glucose due to reversible interactions between ferrocene (Fc)-terminated pluronic and β-cyclodextrin polymer [81]. The micelles transitioned from gel to sol by the temperature response of pluronic or the oxidation of Fc to Fc^+^ resulting in disruption of the Fc-β-CD inclusion complexes. When glucose oxidase capable of reducing glucose to the oxidizing agent hydrogen peroxide was entrapped in the micelles, the complex was capable of undergoing a sol–gel transition over 10 min of exposure to a 20% glucose solution. This system could be utilized as a glucose sensor or to help regulate blood glucose levels by controlled release of drugs.

### Tissue engineering

Tissue engineering employs the paradigm of cells, biomaterials and growth factors to promote regeneration of diseased or defective tissue. In addition to encapsulation of cells, the biomaterial may also elicit the regeneration of new tissue via the migration of existing cells [82]. Cellular adhesion onto the material is a key to successfully incorporating the material into the body [83]. Upon adhesion, cells must be allowed to proliferate and enhance further cell migration in order to repair defective tissue. This process can be hindered by several factors, including protein adsorption, which may create a layer of biofilm. In other cases, fibrous tissue can create a capsule that will compartmentalize the implant from the rest of the body [1]. These processes prevent the effective interaction of the material with its biological surroundings. Several techniques have been developed that can minimize this adsorption, including incorporating PEG onto the surface of the material [84, 85]. However, this treatment and many others may leave the biomaterial biologically inert. It is desired to create a surface available to surrounding cells that is biologically active in order to elicit optimal cellular interaction and adhesion. Surface modification techniques can create a layer on the surface with available functional groups that are known to recruit cells [86].

Tissue engineering scaffolds are three-dimensional, porous structures with surfaces that should be able to mimic biological responses and molecules present in living systems such that living cells may be seeded and proliferate on these supports, then transplanted into a patient [87, 88]. Responsive hydrogels offer highly tunable properties and functionalization through which these responses and molecules can be incorporated into the scaffold structure to promote organized cell growth and architecture.

For example, the innovative work by Okano *et al.* used a culture surface grafted with temperature-responsive PNIPAAm to create a ‘cell sheet’ for tissue engineering applications to overcome poor cell survival and inconsistent cell density observed in cell delivery or some scaffold materials [87]. Cells were seeded on a culture surface treated with hydrophobic PNIPAAm to attach and culture a monolayer of cells, then by reducing the temperature below the LCST of PNIPAAm, the response from a hydrophobic to hydrophilic surface caused the cells detach in a single sheet that could then be implanted in a patient.

**Figure 5.**
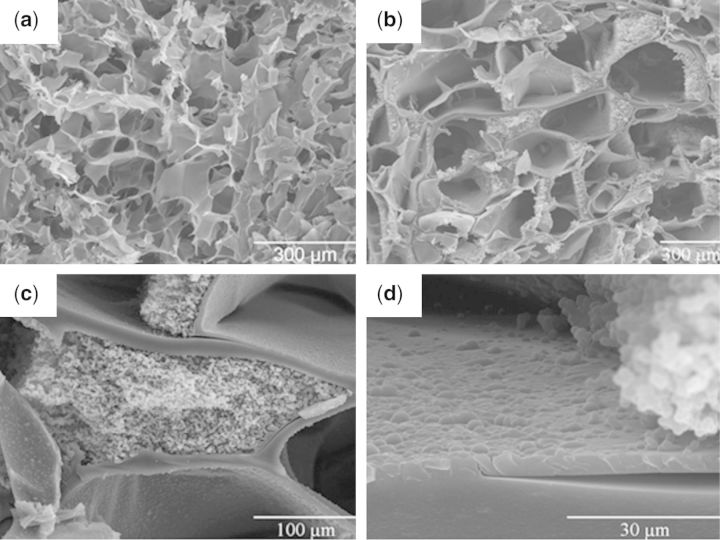
Scanning electron micrographs of chitosan scaffolds (a) native (scale bar = 300 µm) and (b) poly(*N-*isopropylacrylamide)-coated (scale bar = 300 µm); (c) magnified image of coated pores (scale bar = 100 µm) and (d) magnified image of coated pores (scale bar = 30 µm) [89].

Other approaches make use of multi-responsive properties of hydrogels, such as a dual stimuli responsive scaffold to achieve controlled release of growth factors or to promote cell adhesion/detachment [89]. In this case, the chitosan scaffold was coated with PNIPAAm using super critical carbon dioxide, resulting in PNIPAAm coating within the micropores of the chitosan scaffold, maintaining the necessary porous structure for cell growth shown in Fig. 5. The scaffold was infused with a model molecule, and temperature-dependent release of the molecule was confirmed. Additionally, the environmental pH also impacted swelling of the scaffold and release of the molecule. The scaffolds retain the ability to biodegrade following the PNIPAAm coating, a useful property for tissue engineering applications, though there is no indication of the composition of degradation products and the effects on cells.

A material that can capture and release cancer cells in response to glucose concentration and pH was synthesized by grafting poly(acrylamidophenyl boronic acid) brushes on aligned silicon nanowires [90]. At physiological pH, the phenylboronic acid component is known to bind with sialic acid, usually in the terminal position of chains on the cell membrane, enabling the brushes to capture cancer cells expressing these chains. In the presence of glucose at a slightly higher pH, the phenylboronic acid component preferentially bound with glucose, releasing the captured cells. The switch between capture and release was quick, efficient and repeatable without toxicity to the cells. The capture of cells was due both to molecular recognition of the target moieties and topographical interaction between the silicon nanowire array and cell surface. As is, the system is perhaps more useful for detection and assay purposes, though if the pH could be tuned to a more physiologically relevant range, it could have tissue engineering applications as well. Moreover, various cell types could potentially be captured by grafting targeting ligands, molecules or polymers with various responses to the silicon nanowires.

### Self-healing hydrogels

Self-healing hydrogels are capable of partially or completely reforming broken bonds in a spontaneous manner to restore their original structure following fracture or deformation. Especially in combination with multi-responsive properties, self-healing hydrogels have tremendous potential to produce adaptive, biomimetic materials for use in tissue engineering and other biological applications [91].

Complexes that can spontaneously form between peptides or amino acids and transition metals, known as metallo-gelators, can be used to form self-healing hydrogels, as shown by Basak *et al.* [92]. A series of tyrosine-based amphiphiles formed hydrogels in the presence of an aqueous solution of Ni^2+^ ions. The self-healing property of these gels was tuned by varying the chain length of the amphiphiles. The gels were responsive to pH, becoming unstable at pH values >8.0 or <7.0. The gelation temperature of the material was affected by the Ni^2+^ concentration, increasing with greater metal concentrations. Upon application of mechanical force such as shaking, the material was transformed to a solution but returned to a gel state following removal of the force at room temperature, which could have an interesting application as an injectable material though metal incorporation may not be favorable for a biomaterial due to potential toxicity.

In another interesting self-healing application, researchers created a material with an antifouling surface by grafting poly(ethylene oxide) (PEO) on the surface as well as the inside of poly(2-vinyl pyridine) films, calling this ‘3D’ grafting [93]. Since the antifouling property of PEO is known to decrease over time due to oxidative degradation and hydrolytic dissociation of the ester bonds in the polymer, this material possessed novel ability to replace degraded PEO chains on the surface of the film with chains from the interior, thereby healing itself. The relocation of interior chains was driven in theory by a gradient in chemical potential. In practice, the three-dimensional grafted films of shorter chain length were shown to have four-fold longer resistance to protein adsorption than the film grafted with longer chains. The films also exhibited a pH response that was independent of the spontaneous rearrangement of grafted chains, which means it was able to maintain the antifouling properties while undergoing pH-triggered response. Thus, the material can potentially be implanted and resist protein fouling while simultaneously delivering a therapeutic or other molecule.

Self-healing was also demonstrated in a covalently cross-linked hydrogel by decorating the polymer network with dangling hydrocarbon side-chains possessing functional groups that could participate in hydrogen bonding to ‘heal’ a rupture in the gel [94]. Acryloyl-6-aminocaproic acid precursors with amide and carboxylic functional groups were used to synthesize hydrogels and the self-healing properties were subsequently tested in different environmental conditions. It was found that ruptures in coatings of the material could be repaired in low pH conditions, and the material served as an excellent tissue adhesive in gastric mucosa. This is a unique advance, since the material has both the structural integrity of a cross-linked gel and the ability to self-heal or adhere via functionalized side-chains that could potentially be tuned to response to various environmental conditions.

## Conclusions and Future Perspectives

Currently, multi-responsive hydrogels offer the greatest promise of emulating biological systems due to their hydrophilic nature and tunable responses to physiologically relevant stimuli such as pH, temperature and reductive environments. Additionally, external stimuli such as alternating magnetic field and UV light, both prevalent in the biomedical industry, can be used to trigger a response in the material. The responsive properties of these polymers have been combined in nearly every conceivable arrangement to develop innovative drug delivery and tissue-engineering systems. Although reports of dual- and multi-responsive hydrogels have shown a marked increase in recent years, challenges remain in terms of reducing synthesis complexity and ensuring excellent biocompatibility. In the not-so-distant future, we can expect to see the advancement of self-healing hydrogels, biomimetic scaffolds and closed-loop drug delivery systems that are sustainable over long periods of time, resulting in regenerative treatments and implants with improved efficacy to restore a high quality of life for patients.

## Funding

Preparation of this review article was supported in part by the National Science Foundation Graduate Research Fellowship Program [DGE-1110007 to J.M.K.].

*Conflict of interest statement*: None declared.
